# Evolution of MHC class I genes in the endangered loggerhead sea turtle (*Caretta caretta*) revealed by 454 amplicon sequencing

**DOI:** 10.1186/1471-2148-13-95

**Published:** 2013-04-30

**Authors:** Victor A Stiebens, Sonia E Merino, Frédéric J J Chain, Christophe Eizaguirre

**Affiliations:** 1 Department of Evolutionary Ecology of Marine Fishes, GEOMAR|Helmholtz Center for Ocean Research, Kiel, 24105, Germany; 2 National Institute for Development and Fishery (INDP), Mindelo, Cape Verde; 3 Department of Evolutionary Ecology, Max Planck Institute for Evolutionary Biology, Ploen, 24306, Germany

**Keywords:** Major histocompatibility complex, Loggerhead sea turtle, Trans-species polymorphism, Reptiles, Intermediate diversity

## Abstract

**Background:**

In evolutionary and conservation biology, parasitism is often highlighted as a major selective pressure. To fight against parasites and pathogens, genetic diversity of the immune genes of the major histocompatibility complex (MHC) are particularly important. However, the extensive degree of polymorphism observed in these genes makes it difficult to conduct thorough population screenings.

**Methods:**

We utilized a genotyping protocol that uses 454 amplicon sequencing to characterize the MHC class I in the endangered loggerhead sea turtle (*Caretta caretta*) and to investigate their evolution at multiple relevant levels of organization.

**Results:**

MHC class I genes revealed signatures of trans-species polymorphism across several reptile species. In the studied loggerhead turtle individuals, it results in the maintenance of two ancient allelic lineages. We also found that individuals carrying an intermediate number of MHC class I alleles are larger than those with either a low or high number of alleles.

**Conclusions:**

Multiple modes of evolution seem to maintain MHC diversity in the loggerhead turtles, with relatively high polymorphism for an endangered species.

## Background

All organisms are confronted with diseases, which can be particularly threatening to endangered species that show reduced genetic diversities [1]. In vertebrates, growing evidence suggests that genetic diversity is especially important at the level of the major histocompatibility complex (MHC, [2-4]). Since the primary function of MHC molecules is to present parasite-derived peptides to T-lymphocytes, it has been argued that parasites and pathogens are major selective pressures acting on the evolution of MHC genes [1,2,5,6]. There are two main types of MHC molecules, class I and class II. Both classes of molecules function as shuttles that transport peptides from the cytoplasm and display them on the cell surface. MHC class I molecules in particular are expressed by nearly all cell types and present peptides that are derived from proteins degraded by the proteasome [7].

The MHC polymorphism is especially high in the region that encodes for the peptide-binding domain. The residues of the α1 and α2 domains of the MHC class I molecules form the peptide-binding region. Antigenic peptides are anchored at specific residues called antigen binding sites, which are commonly found to be evolving under positive selection in natural populations (e.g. [8]).

The polymorphism present at the MHC genes has regularly been investigated at multiple levels of organization. Firstly, a very particular feature of MHC genes is the existence of trans-species polymorphism (TSP) which has been observed in various taxa (e.g. [9-12]. TSP can occur either through allelic lineages being maintained over long periods of time across speciation events [13,14] or through convergent evolution presumably due to similar parasite pressures [15,16]. Secondly, genetic diversity at MHC loci has been used to measure the immunological fitness of wild populations [1]. Although a direct link between pathogen-mediated population decline and low MHC variation has been difficult to demonstrate in natural populations [17], several studies have reported decreased pathogen resistance among MHC homozygotes (reviewed in [5]). Thirdly, at the individual level, MHC diversity has been associated with numerous fitness traits such as secondary sexual ornamentations [18,19], parasitism [20,21], and life time reproductive success [22]. Although patterns are not clear, several studies have found fitness advantages in individuals carrying either an intermediate number of MHC alleles [20,22-24] or a maximum number of alleles (Heterozygote advantage – [21,25,26]).

Despite a tremendous research effort to understand the evolution of MHC genes and their relevance for conservation biology, surprisingly few studies have focused on the group of non-avian reptiles. The best-characterized MHC example in this taxa is that of the Tuatara in which the second exon of the MHC class I is comprised of two sets of duplicated alleles in most individuals [26,27].

In this study, we used 454 deep amplicon sequencing to investigate the variation of the MHC class I alpha-1 heavy chain in a population of the loggerhead sea turtle (*Caretta caretta*) nesting at the Cape Verde archipelago. Next generation sequencing offers new tools to characterize extreme variation within and between individuals. The use of individually barcoded primers during amplification allows the sequencing of PCR products derived from hundreds of individuals in a single 454 experiment, even for dense gene complexes [28-30]. The read length of 454 sequencers also permits coverage of the entire polymorphic exons of the MHC.

The Cape Verde population of loggerhead turtles is the second largest in the Atlantic [31]. Recently, Monzon-Arguello *et al.*[31] revealed the significant genetic divergence between the Cape Verde rookery and other Atlantic and Mediterranean rookeries. Furthermore, Stiebens et al. [32] showed strong signs of philopatry at the island level, suggesting a complex structure of the rookery with independent colonies. Additionally, in Cape Verde, the fungus *Fusarium solani* was found to be the cause of infections in turtle eggs that accounted for over 80% of mortality in a challenged experiment [33], supporting the need to characterize immune relevant genes.

In this study, after characterizing the MHC class I α genes in the loggerhead turtle, we investigate different modes of evolution at different levels of organization from species to individuals.

## Results

### Phylogeny of MHC genes in reptiles

**Figure 1:**
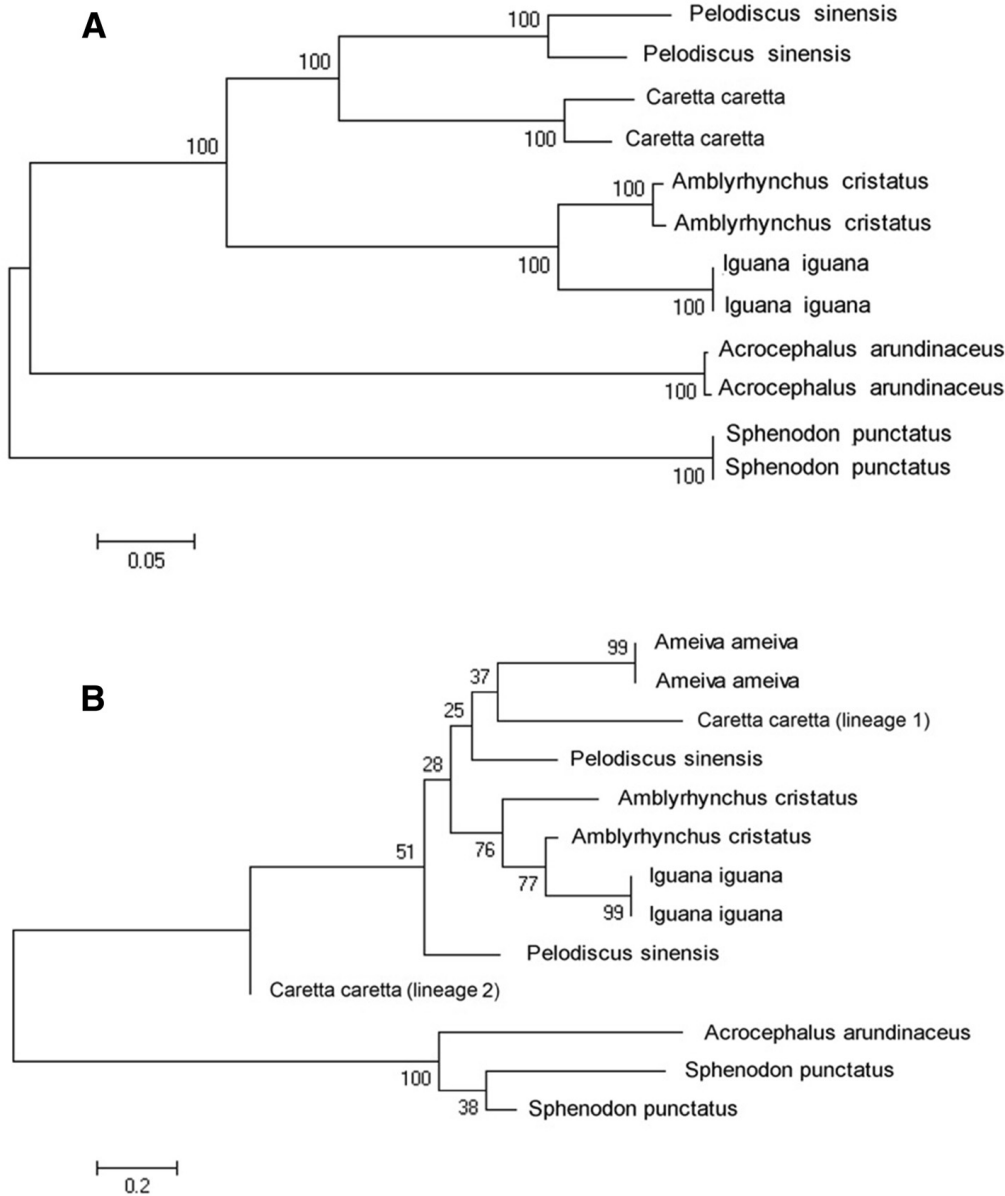
**Neighbor-joining trees.** (**A**) based on the mtDNA control region of five reptile and one bird species. (**B**) based on the MHC class I based on the same species ( Node values (in %) are obtained from 1000 bootstraps). Although (**A**) shows species clustered together, (**B**) demonstrates trans-species polymorphism of the MHC class I gene in reptiles.

To investigate the phylogenetic coherence between neutral and adaptive markers, we built two phylogenetic trees of reptiles using i) mtDNA control region and ii) MHC class I α genes. The trees suggest different evolutionary scenarios (Figure 1A & B). On the one hand, the mtDNA control region clearly separates reptile species where each node is supported by high bootstrap values. In contrast, the MHC class I phylogeny is much weaker and mainly separates the outgroup and the Sphenodon MHC sequences. Interestingly, the loggerhead turtle shows MHC alleles that display closer allelic relationships between species than within species - suggesting trans-species polymorphism over a large range of reptile species and/or a duplication event prior to speciation.

### Phylogeny of MHC in the Cape Verde rookery

**Figure 2:**
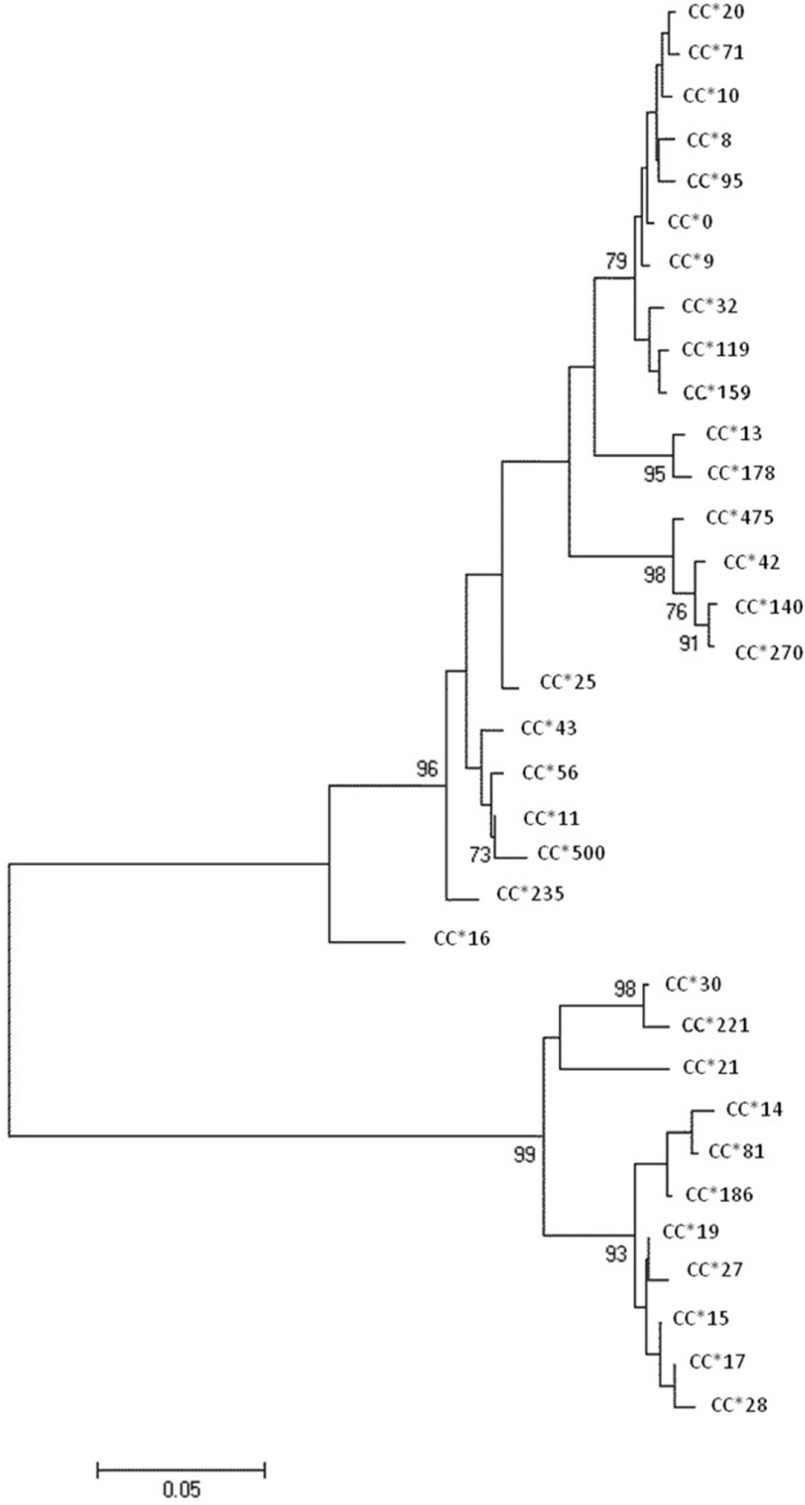
**Neighbor-joining tree based on 1000 bootstraps for all 34 MHC alleles detected.** Three main lineages supported by high bootstrap values are found suggesting gene duplication and/or maintenance of old allelic lineages. Node values are given in percentages, only values higher than 70% are represented.

The phylogenies within the loggerhead turtle population from Cape Verde based on mtDNA and MHC class I alleles were also discordant. For the mtDNA, we found two strong clusters arising from the presence of an extremely divergent haplotype (CC2 in ACCSTR, http://accstr.ufl.edu/resources/mtdna-sequences/) that differs from the other haplotypes from a maximum of 35 point mutations (Additional file 1: Figure S1). As expected from the reptile phylogeny, the MHC neighbor-joining tree identified two main lineages supported by high bootstrap values (Figure 2), which suggests at least one duplication event and/or the maintenance of old allelic lineages. No particular link could be identified between the two phylogenetic trees.

### MHC allelic pool

**Figure 3:**
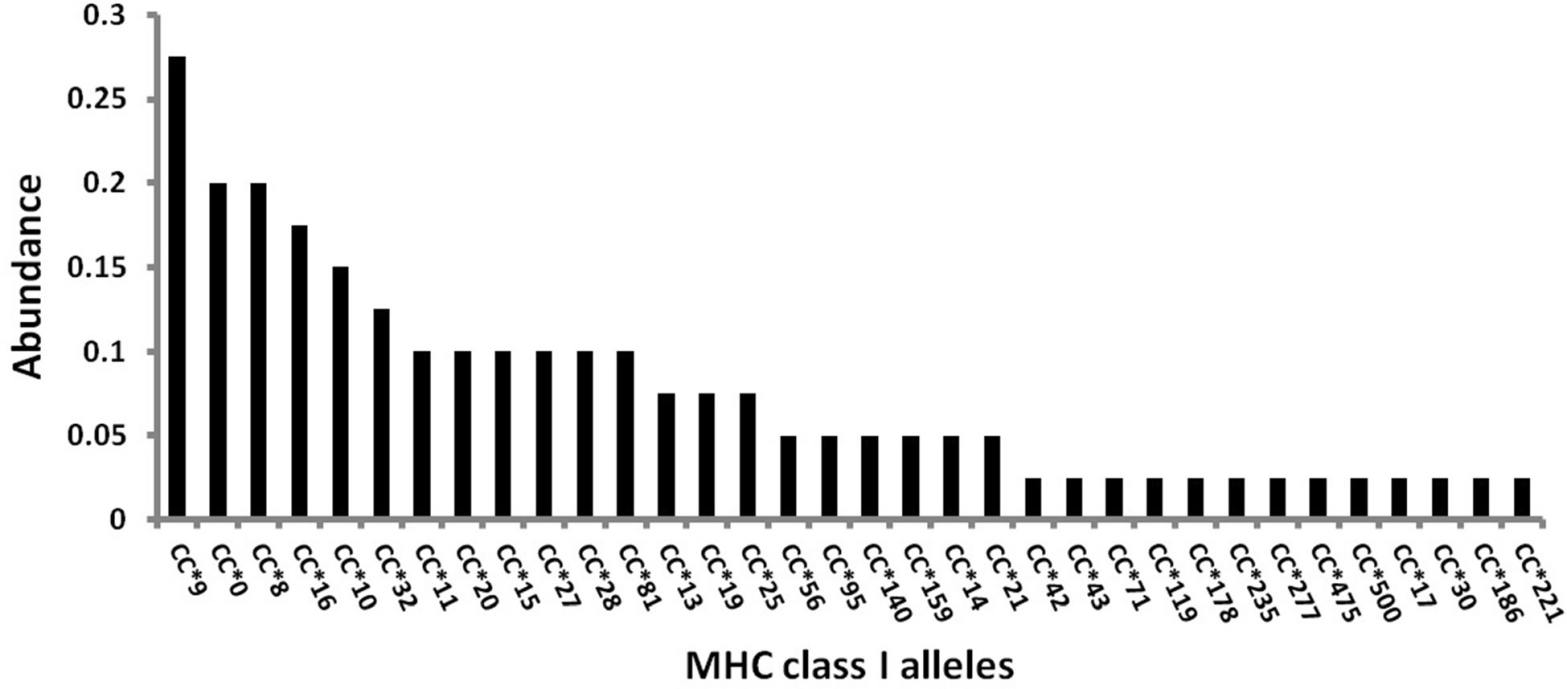
**Histogram representing the frequencies of the 34 alleles found in 40 loggerhead sea turtles.** Thirteen alleles are found only once while 12 occur with a proportion equal to or higher than 10%.

For the 40 turtles sequenced in this study, we obtained approximately 4100 usable 454 reads. After data filtering (see Methods), 34 different alleles were detected with coverage depths varying between 54 and 106 reads per allele (accession numbers: KF021627-KF021666). Allele abundances within the population varied from 0.025 to 0.275 (Figure 3). We found 12 singleton alleles (i.e. found in one individual), but all alleles were present in both independent PCR reactions.

**Figure 4:**
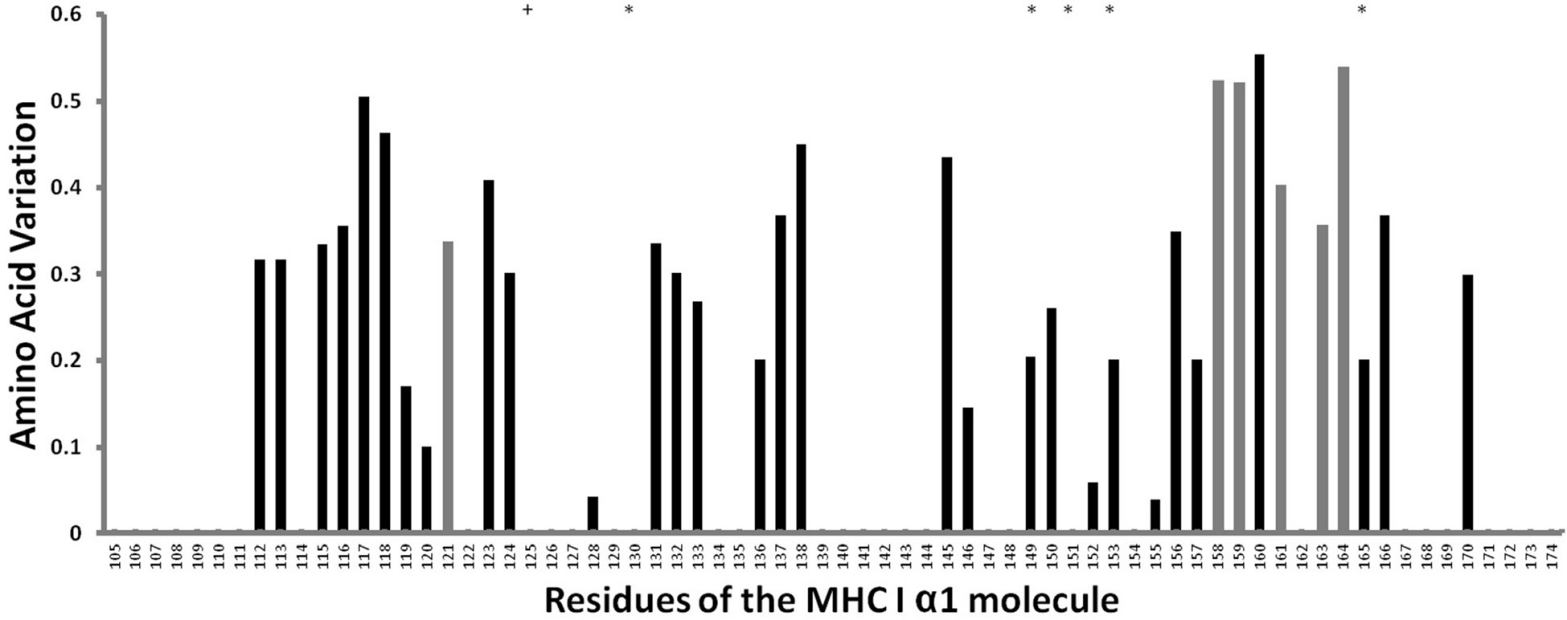
**Variation of amino acid residues in exon 2 of the MHC I gene encoding for the α1 chain of the MHC molecule.** Residues of the 27 alleles detected are given following the reading frame in [12]. The variation for each residue is based on the number and frequency of substitutions and is calculated as y = 1-Valdar01 score, as determined with the Scorecons server by [35]. Grey bars represent sites predicted to be under positive selection, * denotes predicted conserved peptide-binding residues of antigen N and C termini, + denotes predicted salt bridge-forming residues. Primer positions have been removed.

Out of the of 216 basepair (bp) sequence, bp differences ranged from 1 to 69 with a median of 18 (mean = 34.42 +/− 9.89 bp), and from 1 to 32 amino acid changes (median of 11, mean = 16.64 +/− 5.65, Additional file 1 document 2). As would be expected under parasite-mediated balancing selection [34], MHC genes in turtles show strong signs of positive selection: Z = 1.983, p = 0.025. Likelihood ratio tests also suggest that several codon sites in the MHC class I gene are evolving under positive selection (Table 1, Figure 4).

**Table 1:** Table summarizing codon-based tests for positive selection

**Likelihood models**	**Statistical test**	**P-value**	**Estimate for** ω **>1**	**Proportion of sites** ω **>1**	**Codon sites** ω **>1**
M1a vs M2a	18.212	0.00011	3.41529	0.20074	121,158,159
M7 vs M8	23.862	6.58E-06	3.02509	0.26839	121,158,159,161,163,164
M8a vs M8	15.922	3.30E-05			

ω = (d*N /* d*S*) where d*N* represents the number of non synonymous substitutions and d*S* the number of synonymous substitutions. Numbers of codon sites refer to position in the reading frame.

None of the alleles that appeared in more than one individual were in linkage disequilibrium with one other (p = 0.649).

The Hudson four-gamete test [36] implemented in DnaSP [37] detected eight recombination events (RM). These values indicate the minimum number of recombination events in the history of the samples.

Within the 34 alleles found, GENECONV analyses detected six fragments significantly involved in gene conversion events. In addition, the numbers of pairwise internal fragments exceeded the random-assumption of 5% (here, 15.9%) suggesting the occurrence of gene conversion in turtle MHC class I genes.

### Individual MHC allele variation

Individual diversity ranged from one to four alleles (median = 2), indicating the presence of up to four MHC class I loci in this loggerhead population. Out of 7 individuals for which cloning was also performed, 6 genotypes were identical between cloning and 454 sequencing. For the remaining individual, one allele was missing in the cloning approach, but increasing the number of sequenced clones a-posteriori revealed the presence of this allele in this individual (Additional file 1 document 3).

**Figure 5:**
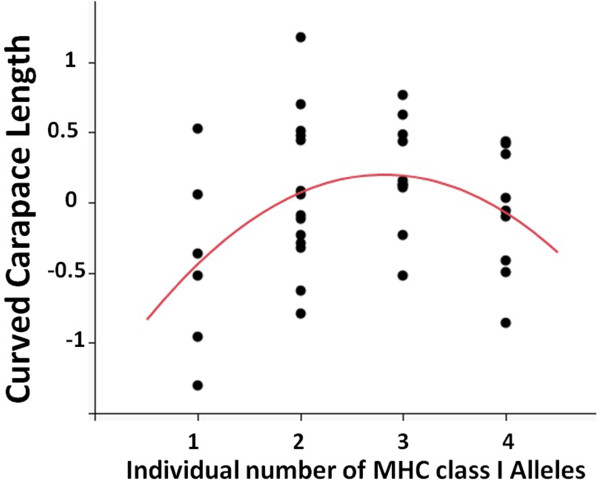
**Relationship between body condition as fitness proxy and individual MHC class I diversity.** Intermediate numbers of individual MHC diversity are associated with higher curved carapace length (CCL) - a proxy of reproductive success. (N = 40, CCL = −0.194 (#alleles)^2^ + 0.091#alleles-0.0413, R^2^ = 0.157).

Identifying fitness proxies in sea turtles is difficult but numerous studies have found that larger turtles have higher clutch size [38,39]. Thus, we used individual size (by using the residuals of the correlation between the curved carapace length and the curved carapace width) as an estimate of turtle body condition. We found that turtles with intermediate MHC diversity were larger than turtles with either higher or lower number of MHC alleles (Quadratic term, Estimate = −0. 194, St.Err. = 0.082, t-value = −2.38, p = 0.023, Figure 5) – suggesting an evolutionary advantage to intermediate MHC diversity.

## Discussion

In this work, we characterized the allelic diversity for genes of the major histocompatibility complex in the endangered loggerhead sea turtle (IUCN 2007). Loggerhead turtles are confronted with multiple direct and indirect anthropogenic threats menacing their genetic diversity – a crucial component of population viability [1]. The MHC genes are not only good estimators of genetic diversity but also play important roles in the onset of the adaptive immune system [5,6]. Here, we used high-throughput genotyping to assess MHC adaptive genetic diversity. Despite the numerous advantages of using next generation sequencing, 454 amplicon sequencing is particularly prone to sequencing errors such as homopolymers [40,41] resulting in an increased frequency of indels [29] or to increased number of sequenced chimeras [34]. Nonetheless, the consequences of such effects can be diminished by combining precautionary PCR preparation (reconditioning steps), independent replicate reactions (using differently labeled primers), accurate primer design [42,43] and sufficient depth of sequencing coverage [34]. Following all those recommendations we were able to address the evolutionary history of MHC class I genes in the endangered loggerhead turtles at multiple evolutionary levels.

Firstly, at a large taxonomic range, we found clear species clustering for the mtDNA control region. Even though the mode of inheritance and the evolutionary rates of both mtDNA and MHC markers are different, contrary to the neutrally evolving mtDNA marker, the MHC genes showed a closer relationship between species than within loggerhead turtle alleles suggesting the existence of TSP within the reptile taxa. TSP corresponds to the maintenance of allelic lineages that are passed on during speciation events to each of the newly formed species [44]. TSP has been reported in related iguana species [12], and our results suggest that TSP spans an even larger taxonomic range, which may arise from the slow evolutionary rate of the basal class of reptiles [45] and/ or via long-term balancing selection.

Given the observed signature of TSP, it was therefore not surprising to find that the sequenced MHC alleles in the loggerhead turtle population from Cape Verde clustered into 2 groups supported by high bootstrap values. Genotypes with such diverse MHC alleles are expected to bind more dissimilar antigens that could then favor their maintenance on en evolutionary time scale [44]. Interestingly, several hypotheses have been proposed to explain the maintenance of MHC polymorphism, but, given the function of these genes, parasite-mediated balancing selection is the most likely driving force (reviewed in [5,6]) as recently shown experimentally [46]. The exceptional allelic diversity usually observed in natural populations, both in terms of the number of specific alleles as well as in terms of amino acid diversity, provides the potential to adapt to a given parasite spectrum.

In the sequenced turtles, we found 34 different alleles suggesting that the MHC class I diversity in the endangered loggerhead turtle is not particularly low compared to other endangered species such as the Namibian cheetah [47] or the European Bison [48]. From a conservation perspective, the fact that numerous individuals carry a unique allelic repertoire indicates the importance of preserving this diversity. Furthermore, our results show that turtles possess up to 4 different MHC alleles, suggesting at least one event of duplication. Since the number of functional MHC loci in the genome represents the bottleneck for adaptation to parasites and pathogens, it might be selectively advantageous to retain duplications at these loci [49]. On an evolutionary time scale, the number of loci within a species is not fixed and may vary over time in a birth-and-death process of gene duplications and deletions [13,50].

It is also worth noting that we found evidence for MHC class I amino acid sites evolving under positive selection. This further supports the view of balancing selection also acting on MHC evolution in turtles. With our dataset, we not only tackled the puzzling evolutionary question of the maintenance of MHC polymorphism but also showed that gene conversion and recombination between copies exist - both playing a role in the generation of high allelic polymorphisms [51,52]. Recombination between loci may explain the occurrence of sequence variants that are particularly divergent, which may then provide particular advantage against parasitic attack. Since many classical MHC genes occur as clusters of functionally intact, duplicated genes, interlocus recombination through unequal crossing-over may also generate sequence polymorphism [53].

Finally, with our dataset we were also able to investigate the relationship between individual MHC diversity and a fitness relevant trait. Identifying relevant fitness traits is complex in marine turtles as reproductive success cannot be followed over generations. Numerous studies, nonetheless, have found that larger turtles achieve a higher clutch size e.g. [38,39]. Here, our results suggest that individuals with an intermediate MHC diversity were larger than those with either high or low diversity. Several studies have reported a relationship between individual MHC diversity and fitness traits, supporting either an advantage for an intermediate diversity [20,24] or for increased heterozygosity [21,25,54]. An intermediate diversity is thought to be due to a combined action of parasite-mediated selection and an excessively strong negative T-cell selection that takes place under high individual MHC diversity [55]. In the case of the loggerhead turtles, up to four MHC alleles seems rather low to trigger increased costs of negative T-cell selection. However the best estimates obtained from mathematical models suggest that such costs can exist with an individual number of expressed MHC molecules in the range of 3 to 25, when combining both MHC class I and class II [55]. This can then apply to the loggerhead turtles. Besides the tropical python, this is the second report of higher individual fitness measure with intermediate MHC class I diversity in reptiles. This correlation may stem from either an advantage of individuals with intermediate MHC diversity being able to better fight off parasites and therefore allocate more energy to growth, or from non-random mortality with regards to MHC. This would result in larger individuals, with intermediate MHC diversity, being older. Both hypotheses are not mutually exclusive but at this stage cannot be disentangled. Another possible explanation is that our data reflect an advantage to heterozygote individuals over homozygotes which would also be predicted by the heterozygote advantage theory (reviewed in [5]). In either case of an optimal diversity or an advantage to heterozygotes, our results suggest an associated cost of homozygosity, a major concern for endangered species such as the loggerhead sea turtle.

## Conclusions

The MHC class I data presented here can serve as an important launching point for studies of conservation genetics, particularly with regard to disease resistance/susceptibility in the loggerhead turtle and other endangered species. Over the last two decades, the MHC has emerged as a valuable complex of genes for evaluating the relative influence of natural selection versus drift and migration on the levels of genetic variation in populations. This is important when considering that selection and adaptation may have its greatest effect on functionally important genes, including genes affecting resistance to pathogens. Evidence for natural selection of the MHC in the loggerhead turtle adds additional insights into the evolution of this gene complex in a phylogenetically basal lineage and demonstrates the potential importance of MHC in the sustainability of an endangered population.

## Methods

### Sampling

Tissue samples from 40 nesting loggerhead sea turtles were collected between July and September 2010 on the island of Sal, Cape Verde. A 3 mm sample was taken from the superficial part of the non-keratinized skin of the flippers using a single-use disposable scalpel immediately after egg deposition. Samples were individually preserved in ethanol until DNA extraction.

### DNA extraction

All tissues were washed in Milli-Q water for 1 minute and were air dried for 15 minutes. DNA extraction was performed using the DNeasy® 96 Blood & Tissue Kit (QIAGEN, Hilden, Germany). All steps followed the manufacturer’s protocol with the exception of the elution, which was conducted in two steps of 100 μl, re-using the first elution.

### mtDNA sequencing

In order to compare the MHC based phylogeny with a phylogeny obtained from a neutral maker, we amplified 723 bp of the mtDNA control region for all individuals using LCN15382 and H950 primers [56]. After amplification and cleaning of PCR product using EXoSap, sequences were loaded into an ABI 3730 Genetic Analyzer (Applied Biosystems, Darmstadt, Germany). For more details, see Stiebens et al. [32]. Four different haplotypes were found: CcA1.3, CcA17.1, CcA17.2 and CcA2.1 following the Archie Carr Center for Sea Turtle Research nomenclature (http://accstr.ufl.edu/resources/mtdna-sequences/).

### MHC primer design

In order to design primers to characterize the highly polymorphic MHC class I exon 2, GeneBank was searched for MHC sequences of related species to the loggerhead turtle. Reptile and avian MHC class I sequences were aligned using BioEdit version 7.0.5.3 [57] and consisted of sequences from reptiles *Malaclemy terrapin* (Genebank accession numbers: GQ495891.1), *Pelodiscus sinensis* (AB185243.1 and AB022885.1), *Sphenodon punctatus* (FJ457094.1, FJ457093.1), and a bird species *Gallus gallus* (AY123227.1). Within this alignment, conserved regions in the exon 2 were selected to design several primer pairs. The exon 2 was chosen because it encodes for a part of the peptide-binding groove involved in parasite recognition. After various PCR tests for the best primer combination, Cc-MHC-I-F (5’-GATGTATGGGTGTGATCTCCGGG-‘3) and Cc-MHC-I-R (5’-TTCACTCGATGCAGGTCDNCTCCAGGT-‘3) showed consistent amplification of multiple MHC class I sequences across several cloning procedures. Although, the Cc-MHC-I-R primer shows polymorphism from the 16^th^ to 18^th^ base pair, no better primers could be designed.

### MHC amplification, cloning, and sequencing

To reduce the risk of PCR artifacts, two independent 20 μl PCR reactions were prepared. Each “replicate” consisted of 2 μl 10× Dreamtaq® Buffer, 1 μl dNTP’s (10 mM), 2 μl of each primer (5pmol/μl), 0.2 μl Taq Polymerase (Dreamtaq^®^) and 2 μl template DNA [~20 μg/μl]. Thermal profile started with an initial denaturing step at 95°C for 3 minutes, followed by 30 cycles of 30 seconds at 94°C, 30 seconds at 66°C and 1 minute at 72°C. The final elongation was set for 5 min at 72°C. The volumes of both reactions were then pooled, of which 30 μl was loaded in an agarose gel (1.5%, 5 h at 45 V). This procedure was recommended by [43] and [58] in order to reduce PCR artifacts. Bands of the expected size (~220 bp) were excised.

Gel purification followed manufacturer’s protocol for the NucleoSpin Extract II Kit (Macherey-Nagel, Düren, Germany). PCR amplicons were cloned with the Qiagen® PCR cloning Kit (Qiagen, Hilden, Germany). The manufacturer’s ligation protocol was followed, except that the ligation-reaction-mixture consisted of 1 μl pDrive Cloning Vector, of 5 μl Ligation Master Mix and of 4 μl PCR products. The transformation protocol was modified as follows: 5 μl of the ligation-reaction mixture were mixed with 25 μl competent cells. Reactions were then heated for 40 seconds at 42°C. Later, 150 μl SOC medium were added and to allow recombinant growth for Kanamycin selection, the reaction mixture was first incubated for 30 minutes at 37°C (slightly shaken) and then plated on a Kan® IptgX-Gal plate. Plasmids were extracted with the Invisorb® Spin Plasmid Mini Two Extraction Kit (Invitek, Berlin, Germany) as described in Kit’s provided protocol, with a final elution step of 50 μl. Cycle sequencing took place in 10 μl PCR reactions consisting of 1 μl Big Dye® Buffer, 1 μl Big Dye® Terminator, 1 μl of the universal M13 Forward primer, 3 μl of HPLC water and 4 μl of extracted plasmid template. The thermal cycling protocol had a first step for 1 minute at 96°C, then 26 cycles at 96°C for 10 seconds and 50°C for 5 seconds. The elongation final step was set at 60°C for 4 minutes. DNA was precipitated and re-diluted in HiDi before being loaded on an ABI 3130 Genetic Analyzer (Applied Biosystems, Darmstadt, Germany). After comparisons of the different sequences obtained with the different primer pairs, the best combination (i.e. the one providing most sequences) was used for high throughput sequencing on a next generation sequencing platform.

### Barcoded 454 sequencing of MHC genes

The 454 next generation sequencing platform using a barcoded deep amplicon approach [29,30] was chosen because of the long sequence reads and large coverage to help determine high intra and inter individual variability. To this end, DNA concentrations were standardized to 10 ng/μl in order to maximize the likelihood of equal coverage of all samples. As previously described, two independent PCR reactions were performed. For each replicate, the protocol was split into two steps. In the first step, PCR conditions were kept as described above, but the number of PCR cycles was reduced to 25. The first PCR products were used as a template for another 10 PCR cycles. The reconditioning procedure coupled with independent PCR reactions reduces the final proportion of artifacts [42], a major problem with new sequencing technologies. The reconditioning step used 454 sequencing adaptors (Forward side TitaA CCATCTCATCCCTGCGTGTCTCCGACTCAG; Reverse side TitaB CCTATCCCCTGTGTGCCTTGGCAGTCTCAG, GATC, Constance, Germany), followed by a 10 nucleotide individual tag (MID, Roche) and the newly developed MHC class I primer pair. The MID tags were designed such that the random accumulation of up to two polymerase errors in the MID would still lead to the correct individual identification. For a given individual, replicated PCRs had the same forward MID tags but different reverse MID tags which allowed us to track the product of each PCR reaction all along the amplification and sequencing.

After amplification, amplicons were cleaned using the Qiagen PCR Purification Kit (Qiagen, Hilden, Germany). The cleaned products were run on gels, to verify the presence of the expected bands. From all cleaned samples, DNA concentration was re-measured and all samples were pooled so that each PCR reaction contributed to an equal amount of 100 ng/sample. To remove potential unspecific amplicons, the final pool was loaded on a 1.5% agarose gel (14 h at 30 V). Bands of ~340 bp were cut out and products were extracted as described above.

### Individual MHC genotyping

MHC alleles were called and assigned to each individual using Perl scripts. Reads were screened for the forward and reverse sequencing primers, allowing one nucleotide mismatch or indel (insertion/deletion) in case of sequencing errors and otherwise discarded. Remaining reads were then assigned to individuals based on MID tags, again allowing for one nucleotide mismatch or indel. Reads were then trimmed (removing the primer and MID sequence) and aligned using BioEdit, resulting in a set of putative allele variants for each individual. To cull out less reliable sequence variants, alleles were retained only if they met the following criteria per individual: (1) if they appeared in both independent PCR preparations (both MID tags) and (2) if their frequency (in terms of proportion of reads) was above 10% of the most frequently occurring allele within that individual. The remaining variants, although they might stem from different loci, are referred to as “alleles” and make up our final allele dataset.

Errors occurring during the 454 sequencing include substitutions and small indels [29,30], and these were expected to occur randomly across the sequence. From our MID tags, the frequency of errors resulting in base substitutions was low. Therefore, the probability of multiple, identical substitution errors is estimated to be low [30]. Single-base indels occurring in homopolymer tracts were relatively common and were non-randomly distributed along the sequence. However, such variants were removed with our method because of their low frequency of occurrence within an individual and across independent replicate PCR reactions.

### Data analyses

Under positive selection, a relative excess of non-synonymous over synonymous substitutions is expected [59]. We calculated the relative rates of synonymous (d_*S*_) and non-synonymous (d_*N*_) substitutions following the method of Nei and Gojobory [60] with the Jukes-Cantor [61] correction for multiple substitutions implemented in MEGA 4 [62]. The rate ratio d_*N*_/d_*S*_ was tested for significant deviation from one using a Z-test.

MEGA 4 was also used to build a neighbor-joining tree with 1000 bootstraps for all MHC alleles found in the sampled turtles. Two additional neighbor-joining trees were simulated: one based on the control region of the mitochondrial genome (mtDNA) of 6 reptile species and one based on the MHC class I of 5 reptile species.

Maximum likelihood site models implemented in the CODEML program from PAML version 4.4 [63] were used to test for evidence of positive selection and to identify branch-specific positively selected codon sites [ω > 1, where ω = (d_*N*_*/*d_*S*_)]. The maximum likelihood procedures evaluate heterogeneous rate ratios (ω) among sites by applying different models of codon evolution. Three likelihood-ratio tests of positive selection were performed comparing the models M1a (nearly neutral) vs M2a (positive selection), M7 (ß) vs M8 (ß + ω), and M8a (ß + ω = 1) vs M8 [64]. In these likelihood-ratio tests, two nested models are compared: a model based on the null hypothesis of no positive selection, and a model that allows some sites to evolve under positive selection. The null model M1a assumes two site classes in the molecule with 0 < ω _0_ < 1 and ω_1_ = 1 in proportions *p*_0_ and *p*_1_ = 1-*p*_0_. The alternative model M2a incorporates another class of sites with ω_2_ > 1 and the proportion *p*_2_ estimated from the data. The null model M7 assumes a beta distribution for ω, not allowing positive selection (0 < ω < 1). The alternative model M8 has additional classes of sites that allow some codons to evolve under positive selection (ω > 1, [62]). A third null model M8a differs from model M8 in that its additional class of sites are evolving neutrally (ω = 1). In the models M2a and M8, positively selected sites are inferred from posterior probabilities calculated by the Bayes empirical Bayes method [65]. Because MHC alleles are so variable and often represent ancient lineages (TSP), we thought the evaluation of dN and dS appropriate despite the comparison within a species.

We used the ScoreCons online server [35] to determine variation for amino acid residues in the exon 2 of the loggerhead turtles. The software MultiLocus 1.22 [66] was used to estimate linkage disequilibrium between detected alleles using 10000 randomizations.

The minimum number of recombinant events (RM) was calculated after Hudson and Kaplan [four-gamete method, McVean *et al.*[36] using the software DnaSP.

The program GENECONV version 1.81 was used to detect sequence fragments that were likely to have been subjected to gene conversions. GENECONV detects pairs of sequences that share unusually long stretches of similarity given their overall polymorphism [67]. We used global and pairwise permutation tests (10,000 replicates) to assess significance.

Although fitness is difficult to estimate in loggerhead turtles, studies have shown that larger females have a higher clutch size, linking turtle morphometrics to high fecundity [38,39]. As a fitness proxy we used the curved carapace length corrected (residuals of correlation) for curved carapace width, as equivalent to body condition. Residuals for this correlation were then tested against individual number of MHC alleles (linear and quadratic terms) following [20]. Curved carapace length and curved carapace width were measured for all turtles immediately after egg deposition.

## Competing interest

The authors declare no competing interests.

## Authors’ contributions

CE designed the study. VS, SEM, and CE participated in sample collection. VS and CE performed the statistical analyses. FC wrote the bioinformatic scripts. VS, FC and CE drafted the manuscript. All authors read and approved the final manuscript.

## Supplementary Material

Additional file 1: Figure S1 Neighbor-joining tree of the mtDNA control region. All sequences have been deposited on Archie Carr Centre for Sea Turtle Research (http://accstr.ufl.edu/resources/mtdna-sequences/). **Document 2** Amino acid alignment of loggerhead turtle MHC class I alleles. Dots indicate identity with the loggerhead Cc*0 sequence. **Document 3** Table summarizing the genotyping of 7 turtles using two different methods: cloning/sequencing vs. 454 sequencing. Allele identities are given together with the number of clones picked and sequenced for each individual. Row in bold shows a discrepancy between cloning and 454 sequencing. ^$^indicates a posteriori screen. Click here for file
