# Supplementary material for: Evolution of MHC class I genes in the endangered loggerhead sea turtle (Caretta caretta) revealed by 454 amplicon sequencing
Source: BMC Evol Biol. 2013 Apr 30;13:95. doi: 10.1186/1471-2148-13-95 (PMC3655109; doi:10.1186/1471-2148-13-95)
Supplement: Additional file 1: Figure S1 — Neighbor-joining tree of the mtDNA control region. All sequences have been deposited on Archie Carr Centre for Sea Turtle Research (http://accstr.ufl.edu/resources/mtdna-sequences/). Document 2 Amino acid alignment of loggerhead turtle MHC class I alleles. Dots indicate identity with the loggerhead Cc*0 sequence. Document 3 Table summarizing the genotyping of 7 turtles using two different methods: cloning/sequencing vs. 454 sequencing. Allele identities are given together with the number of clones picked and sequenced for each individual. Row in bold shows a discrepancy between cloning and 454 sequencing. $indicates a posteriori screen. [file 1471-2148-13-95-S1.doc]

**Additional file 1 Figure S1**

Neighbor-joining tree of the mtDNA control region. All sequences have been deposited on Archie Carr Centre for Sea Turtle Research (<http://accstr.ufl.edu/resources/mtdna-sequences/>).

**
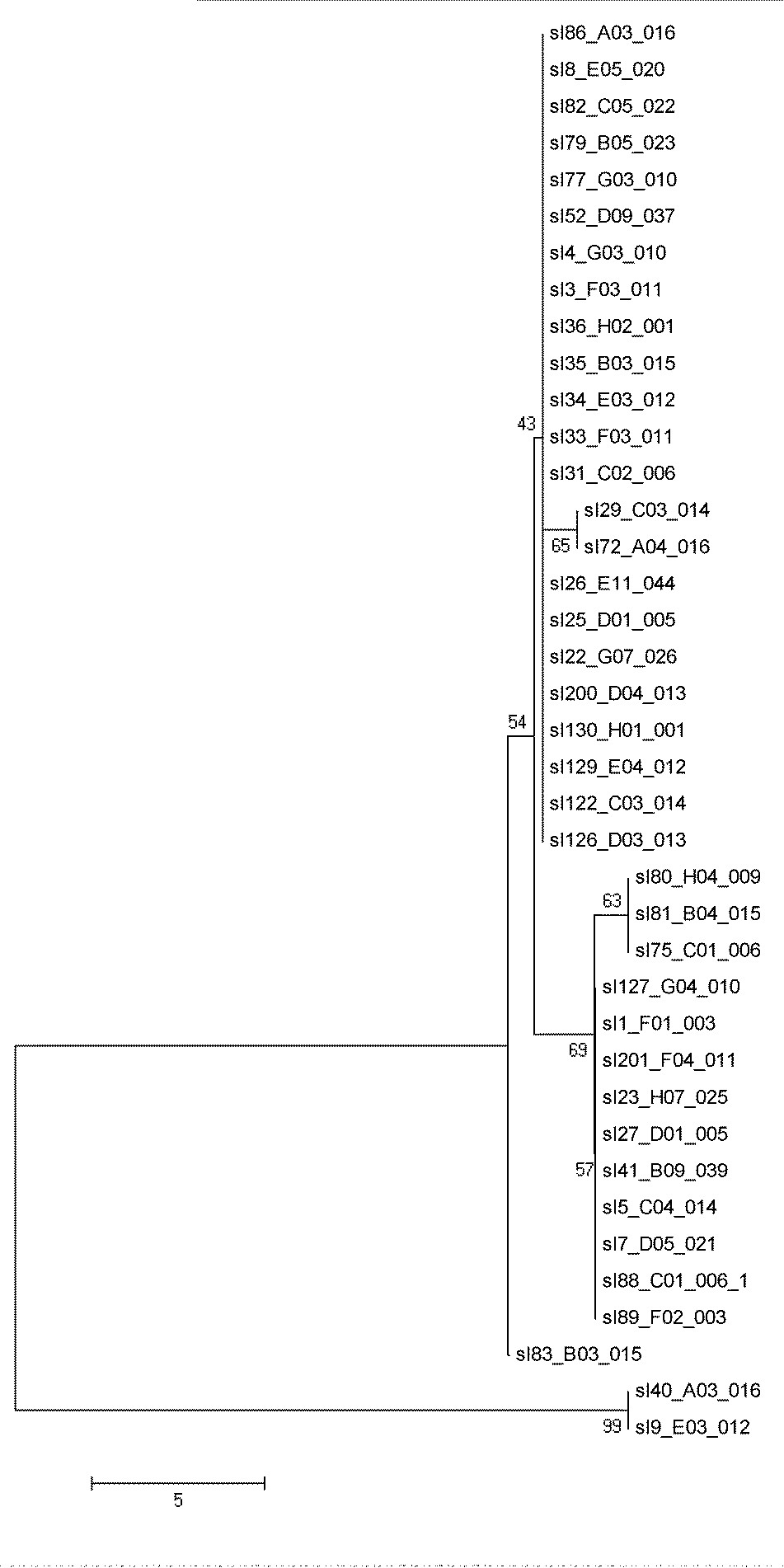
**

**Additional file 1 document 2**

Amino acid alignment of loggerhead turtle MHC class I alleles. Dots indicate identity with the loggerhead Cc*0 sequence.

**110 120 130 140 150 160 170**

**....|....|....|....|....|....|....|....|....|....|....|....|....|....|**

**CC*0 MYGCDLRGGDGSKGGLFQYAYDGRDFVSLDKDRETWVAADDGAQVTKRKWDGDKSIAQTYRAYLEPTCIE**

**CC*8 .................................................................K....**

**CC*9 ...............F......................................................**

**CC*10 .................................................................L....**

**CC*11 ...............FY.........I........................A.RGFT.RQ..........**

**CC*13 ...............F..................................EA.R....GW..........**

**CC*14 .......EE.NTTQVFY.DS......LTF..ETV......I...IS..R..AEVLDN.RWKR........**

**CC*15 .......EE.NTTW.FY.DS......LTF..ETV......I...IS..R..AEVLDN.RWKR........**

**CC*16 .......DD...I..FR.D.......I..............A..I......A.RGFT.RR..........**

**CC*17 .......EE.NTTW.FY.DS......LTF..ETV......I...IS..R..AEVLDN.RWKR........**

**CC*19 .......EE.NTTW.FY.DS......LTF..ETV......I...IS..R..AEVLDN.RWKR........**

**CC*20 .................................................................V....**

**CC*21 .......EE.NVIQ.FY.DS...Q..LTF..ETM......I...I...R..AEVLDN.QWKR........**

**CC*25 ...............F...................................A.RGFT.RQ..........**

**CC*27 .......EE.NTTW.FY.DS......LTF..ETV......I...IS..R..AEVLDN.RWKR........**

**CC*28 .......EE.N.TW.FY.DS......LTF..ETV......I...IS..R..AEVLDN.RWKR........**

**CC*30 .......EE.NVIQ.FY.DS......LTF..ETM......I...I..KR..VEIGDN.RWKR........**

**CC*32 ...............F..........I...........................................**

**CC*42 ............I..FS.........I..............A..I.............RE..........**

**CC*43 ...............FY.........I........................A.RGFT.RQ..........**

**CC*56 ...............FY.........I........................A.RGFT.RR..........**

**CC*71 ...............F.................................................V....**

**CC*81 .......EE.NTTWVFY.DS......LTF..ETV......I...IS..R..AEVLDN.RWKR........**

**CC*95 ...............F......................................................**

**CC*119 ...............F..........I......................................K....**

**CC*140 ............I..FS.........I..............A..I.............RE.....L....**

**CC*159 ...............F..........I......................................M....**

**CC*178 ...............F..................................EA.R....GW.....V....**

**CC*186 .......EE.NTTQVFY.DS......LTF..ETV......I...IS..R..AEVLDN.RWKR........**

**CC*221 .......EE.NVIQ.FY.DS......LTF..ETM......I...I..KR..VEIGDN.RWKR........**

**CC*235 ............I..FS.........I........................A.RGFT.RR..........**

**CC*277 ............I..FS.........I..............A..I.............RE.....V....**

**CC*475 ............I..FS.........I..............A..I.............RQ..........**

**CC*500 ...............FY.........I........................A.RGFT.RQ..........**

**Additional fie1 document 3**

Table summarizing the genotyping of 7 turtles using two different methods: cloning/sequencing vs. 454 sequencing. Allele identities are given together with the number of clones picked and sequenced for each individual. Row in bold shows a discrepancy between cloning and 454 sequencing. ^$^indicates a posteriori screen.

| **Turtle ID** | **Cloning: Allele identity** | **Number**  **of clones** | **454: Allele identity** |
| --- | --- | --- | --- |
| **SL3** | CC*0, CC*2, CC*13 | 33 | CC*0, CC*02, CC*13 |
| **SL4** | CC*4, CC*12 | 29 | CC*04, CC*12 |
| **SL5** | **CC*10, CC*03, CC*08, CC*013^$^** | **24+24^$^** | **CC*10, CC*03, CC*08, CC*013** |
| **SL7** | CC*0, | 31 | CC*0 |
| **SL8** | CC*24, CC*13 | 32 | CC*24, CC*13 |
| **SL9** | CC*10, CC*18 | 29 | CC*10, CC*18 |
| **SL22** | CC*1, CC*3, CC*12 | 34 | CC*1, CC*3, CC*12 |
